# Attempt to assess Canada’s expertise in global health research falls short

**DOI:** 10.1186/s12961-020-00634-5

**Published:** 2020-11-03

**Authors:** Theresa W. Gyorkos

**Affiliations:** WHO Collaborating Centre for Research and Training in Parasite Epidemiology and Control, Department of Epidemiology, Biostatistics and Occupational Health, Faculty of Medicine, McGill University, Purvis Hall, 1020 Pine Avenue West, Montreal, Québec H3A 1A2 Canada

**Keywords:** global health, expertise, Canadian, assessment, research, health policy, funding, rapid environmental scan methodology, underestimation, bibliometrics

## Abstract

The recent article by Nagi et al. (*Health Res Policy Syst* 18:37, 2020) considerably underestimates the size of the global health research community in Canada as well as its geographical distribution, its breadth and depth of experience and expertise, and its overall contribution to addressing the world’s greatest global health priorities. Global health researchers, practitioners, policy-makers, strategists and funders/donors would benefit from a more accurate in-depth and comprehensive analysis.

## Main text

The article by Nagi et al. [1] attempts to assess Canada’s expertise in global health research. Unfortunately, this attempt falls short because of important flaws in all three metrics of its ‘rapid environmental scan methodology’ (i.e. global health research funding inputs, global health research activities and global health research outputs). The use of a ‘rapid environmental scan methodology’ as described in this context is inappropriate and results in misleading conclusions.

Restricting global health funding inputs to funds awarded only by the Canadian Institutes of Health Research disregards the funding success of Canadian global health researchers in competing for millions of dollars in research awards from Global Affairs Canada, the Bill and Melinda Gates Foundation, the National Institutes of Health, the Wellcome Trust, the European Commission, WHO, UNAIDS, the World Bank, and the Global Fund to Fight AIDS, Tuberculosis and Malaria, to name but a few of the major funders of global health research. In 2019 alone, the Bill and Melinda Gates Foundation awarded a total of US$19,194,988 to researchers in six Canadian universities (including their affiliated hospital-based research institutions) in its Global Health programme [2].

Restricting global health research activities to training programmes, Research Chairs programmes and WHO Collaborating Centres disregards other activities in which global health researchers from across Canada play a crucial role (e.g. The Canadian Coalition for Global Health Research (CCGHR), the Canadian Society for International Health (CSIH), the Canadian Network for Neglected Tropical Diseases (CNNTD), and Working Groups and Expert Panels of WHO, to name a few). Mention should also be made of the inaugural listing of over 100 Canadian women, many of them researchers, which was prompted by an initiative of the *Lancet* in recognising the contributions of women in global health [3].

Restricting global health research outputs to PubMed citations using a search strategy that only included ‘Global Health’ as a MeSH heading and author affiliation as ‘Canada’ disregards the enormous contributions made by Canadian global health researchers to the published evidence base of many of the world’s top global health research priorities. For example, using the same time span (i.e. from January 1, 2013, to March 1, 2018), Canadian global health researchers were authors in 1456 peer-reviewed publications on HIV/AIDS, 1251 publications on tuberculosis and 632 publications on malaria (PubMed accessed 10 April, 2020, using the search terms “Canada” and “HIV/AIDS”, “tuberculosis” and “malaria”, respectively), eclipsing the total of 882 publications for all of global health research reported in the Nagi et al. [1] article. It should also be emphasised that the evidence base for key aspects of global health research, as defined by Nagi et al. [1] themselves (e.g. including health systems and health policy, among others) would require a much broader bibliometric analysis than that presented.

## Conclusions

In summary, the Nagi et al. [1] article considerably underestimates the size of the global health research community in Canada as well as its geographical distribution, its breadth and depth of experience and expertise, and its overall contribution to addressing the world’s greatest global health priorities. Global health researchers, practitioners, policy-makers, strategists and funders/donors would benefit from a more accurate in-depth and comprehensive analysis!

## Data Availability

Not applicable.
